# ﻿Two new species and a new record of Hypoxylaceae (Xylariales, Ascomycota) from Mexico

**DOI:** 10.3897/mycokeys.111.133046

**Published:** 2024-12-17

**Authors:** Pamela E. Reyes, Juan Pablo Pinzón, Ricardo Valenzuela, Tania Raymundo, Juan Tun-Garrido, Ricardo García-Sandoval

**Affiliations:** 1 Campus de Ciencias Biológicas y Agropecuarias, Laboratorio de Recursos Florísticos de Mesoamérica, Universidad Autónoma de Yucatán, Carretera Mérida-Xmatkuil Km. 15.5, Plan de Ayala ÌII, Mérida 97100, Yucatán, Mexico Universidad Autónoma de Yucatán Mérida Mexico; 2 Laboratorio de Micología, Departamento de Botánica, Escuela Nacional de Ciencias Biológicas, Instituto Politécnico Nacional, Prolongación de Carpio y Plan de Ayala s/n, Col. Santo Tomás, Miguel Hidalgo, Ciudad de México 11350, Mexico Instituto Politécnico Nacional Ciudad de México Mexico; 3 Facultad de Ciencias, Universidad Nacional Autónoma de México, Av. Universidad 3000, Circuito Exterior s/n, Ciudad Universitaria, Coyoacán, Ciudad de México 04510, Mexico Universidad Nacional Autónoma de México Ciudad de México Mexico

**Keywords:** Diversity, Neotropics, phylogeny, Yucatan Peninsula

## Abstract

The family Hypoxylaceae has a cosmopolitan distribution with greater diversity in tropical regions, its growth habit is saprotrophic, endophytic and potentially phytopathogenic. From the revision of herbarium specimens and field collections from the Yucatan Peninsula, two new species were described: *Annulohypoxylonfusisporum*, characterized by having fusiform spores and *Hypoxylonxmatkuilense* which is distinguished by having stromata vinaceous and dark brown KOH-extractable pigments. The species are described based on morphological characters and Bayesian Inference analyses of four molecular markers (ITS, LSU, RPB2 and TUB2). In addition, one new record from Mexico is presented: *Hypoxylonbellicolor*. The presence of *Daldiniaeschscholtzii*, *H.lenormandii*, *H.lividipigmentum* and *Entonaemaliquescens* is confirmed with molecular data.

## ﻿Introduction

The family Hypoxylaceae (Xylariales, Ascomycota) contains 22 genera and 495 species (Wijayawardene et al. 2022; Bánki et al. 2024) 75 species have been cited from Mexico: *Annulohypoxylon* (6), *Daldinia* (13), *Durotheca* (1), *Entonaema* (4), *Hypomontagnella* (2), *Hypoxylon* (36), *Jackrogersella* (2), *Parahypoxylon* (1), *Phylacia* (8), *Pyrenopolyporus* (2) (CONABIO 2024).

They are characterized by having erect, glomerate, pulvinate, discoid, effused-pulvinate, hemispherical, spherical or peltate stromata; solitary or confluent, brightly colored, dark or black, pruinose or smooth, with or without extractable pigments that are evident with 10% KOH; spherical, obovoid or tubular perithecia with spherical, umbilicate or papillate ostioles, with or without discs formed by dehiscence of the surrounding tissue. They share a nodulisporium-like asexual state (which is one of the features that sets them apart from Xylariaceae) and are distributed in tropical and temperate areas of the world (Ju and Rogers 1996). Their lifestyle is mainly endophytic, saprotrophic and even associated with insect vectors (Wendt et al. 2018).

Among the most important taxonomic works that have been published about the genera that at this moment are considered as part of Hypoxylaceae, we can find the monograph of *Hypoxylon* by Ju and Rogers (1996), the revision of Child (1932) who published the first monograph of *Daldinia* with 11 species, furthermore Ju et al. (1997) described 19 species of the genus. Stadler et al. (2014a) recognized 47 taxa of *Daldinia* based on morphological, chemotaxonomic and phylogenetic evidence; Sir et al. (2016) described *D.korfii* from the Yungas region in Argentina; Chlebicki (2022) presented a review of the genus from Poland.

The genus *Entonaema*, was erected by Alfred Möller (1901) who described to *E.liquescens* and *E.mesentericum*, currently as *Xylariamesenterica* (Stadler et al. 2008). Pošta et al. (2023) accepted six species: *E.liquescens*, *E.cinnabarinum*, *E.dengii*, *E.moluccanum*, *E.globosum*, and *E.siamensis*.

In the study by Wendt et al. (2018), based on analysis with four molecular markers (ITS, LSU, RPB2 and TUB2) Hypoxylaceae was formally recognized, clearly segregated from Xylariaceae with the following genera: *Acrostaphylus*, *Annulohypoxylon*, *Anthocanalis*, *Ascoporia*, *Chlorostroma*, *Daldinia*, *Entonaema*, *Henningsina*, *Hypoxylina*, *Hypoxylon*, *Jackrogersella*, *Phylacia*, *Pyrenomyxa*, *Pyrenopolyporus*, *Rhopalostroma*, *Rostrohypoxylon*, *Ruwenzoria*, *Sphaeria*, *Thamnomyces* and *Thuemenella*.

Lambert et al. (2019) erected *Hypomontagnella* based on a study of *Hypoxylonmonticulosum* and its allies. The features that characterize them are the stromata woody to carbonaceous lacking colored granules, papillate ostioles usually with a black annulate disc, without apparent KOH-extractable pigments in mature stromata and perispores smooth or with transversally striate ornamentations.

Recently, Cedeño-Sanchez et al. (2023) proposed *Parahypoxylon* as a new genus including *P.papillatum* and *P.ruwenzoriense*; Lambert et al. (2023) studied *Phylacia* species in Argentina and found a close relationship with *Rhopalostroma*, *Thamnomyces* and *Daldiniaspecies*, which all have similar secondary metabolites.

In Mexico, 75 species of the family are known on all types of vegetation, standing out the study of San Martín et al. (1999b) in which they reviewed the genus Hypoxylon including some members of Section Annulata (now genus *Annulohypoxylon*); San Martín and Lavin (1997) described three species of *Entonaema*; Medel et al. (2006) made a review of *Phylacia*, recording eight species; Barbosa-Reséndiz et al. (2020) reported an updated list of *Daldinia* in Mexico; Raymundo et al. (2014, 2017, 2021) and Reyes et al. (2020) have reported species of Hypoxylaceae from Protected Natural Areas: Chamela-Cuixmala, Cozumel Island, Lagunas de Chacahua National Park, Sierra de Álamos-Río Cuchujaqui and El Cielo. For this work, several specimens from herbaria and field collections in the Yucatan Peninsula have been studied taxonomically. Morphological and molecular analyses have revealed some taxonomic novelties that we report in this document.

## ﻿Materials and methods

### ﻿Collections sites and sampling

In the current paper, 240 specimens were studied, of which 148 are deposited in the “Alfredo Barrera Marín” Herbarium of the Universidad Autónoma de Yucatán (UADY) and the “Gastón Guzmán Huerta” Fungi Collection of the Herbarium of the Escuela Nacional de Ciencias Biológicas, Instituto Politécnico Nacional (ENCB), while 77 were collected in the Campeche, Quintana Roo and Yucatan States between 2021–2023. In this study, nine specimens were analyzed morphologically and sequenced.

### ﻿Morphological characterization

The description of macromorphological features was carried out with water and 10% KOH to examine perisporium dehiscense and to view the stromatal pigments, Melzer’s reagent to show the amyloid reaction of the ascal apical apparatus, specialized literature in each genus was reviewed (San Martín et al. 1999b; Stadler et al. 2014a; Kuhnert et al. 2016; Lambert et al. 2019; Reyes et al. 2020), colors were described according to Rayner (1970).

### ﻿DNA extraction, PCR and sequencing

The stromata were macerated in liquid nitrogen, then the QIAGEN DNeasy kit (Hilden, Germany) was used according to the manufacturer’s specifications. The primers used were reported by White et al. (1990) (ITS1, ITS4), Gardes and Brunes (1993) (ITS1-F) for ITS (internal transcriber spacer gene); Vilgalys and Hester (1990) (LR07, LR0R) for LSU (large subunit ribosomal gene); Liu et al. (1999) (RPB2-5F, -7cR) for RPB2 (partial second largest subunit of the DNA-directed RNA polymerase II gene); O’Donnell and Cigelnik (1997) (T1, T22) for TUB2 (beta-tubulin gene). PCR reactions were made using 25 µl reaction:12.5 µl GoTaq® (Promega, Madison, USA) “Master Mix”, 2-3 µl DNA, 0.5 µl of each primer (100 µM), BSA 2.5 µl and 7 µl of sterilized water. The PCR conditions are detailed in Table 1. Sequencing was performed by the Sanger technique at Macrogen Inc.

**Table 1.** PCR conditions used in this study.

DNA locus	Initial denaturation	Cycles	Denaturation	Annealing	Elongation	Final denaturation
ITS	94 °C-5 mins	35	94 °C-1 min	55 °C-1 min	72 °C-1 min	72 °C-10 mins
LSU	94 °C-5 mins	34	94 °C- 1 min	52 °C-1 min	72 °C -2 mins	72 °C-10 mins
RPB_2_	94 °C- 5 mins	38	94 °C-30 s	53 °C-1 min	72 °C-1.30 mins	72 °C-10 mins
TUB_2_	94 °C-5 mins	38	94 °C -30 s	47 °C-30 s	72 °C-2.30 mins	72 °C-10 mins

### ﻿Molecular phylogenetic analyses

A concatenated matrix of the four molecular markers was constructed using 274 sequences from 71 species as a reference from the GenBank (Appendix 1) and 36 obtained in the present study. Sequences were aligned using MAFFT software (Katoh and Kuma 2002) and edited in BioEdit (Hall 1999). The evolution model that best fit each group of sequences was obtained using the j Model Test 2.1.10 software (Posada and Crandall 1998) supported by Bayesian Information Criterion (BIC). A Maximum Likelihood analysis was performed in RaxML 8.2.12 (Stamatakis 2006) with 1000 bootstrap replicates. Additionally, a Bayesian analysis was carried out with the same matrix in Mr.Bayes 3.2.6. (Ronquist and Huelsenbeck 2003) using four MCMC chains, 10000000 generations, taking samples every 1000 generations, applying a burnin of 25%, *Xylariapolymorpha* was used as an outgroup.

## ﻿Results

### ﻿Phylogenetic analysis

The molecular matrix of the four concatenated loci (ITS, LSU, RPB2 and TUB2) was 7283 bp in length, having 1640 for the first, 2417 for the second, 1200 for the third and 2026 for the fourth, applying the following substitution models: SYM+I+G for ITS, HKY+I+G for TUB2 and GTR+I+ G for LSU and RPB2.

**Figure 1.**
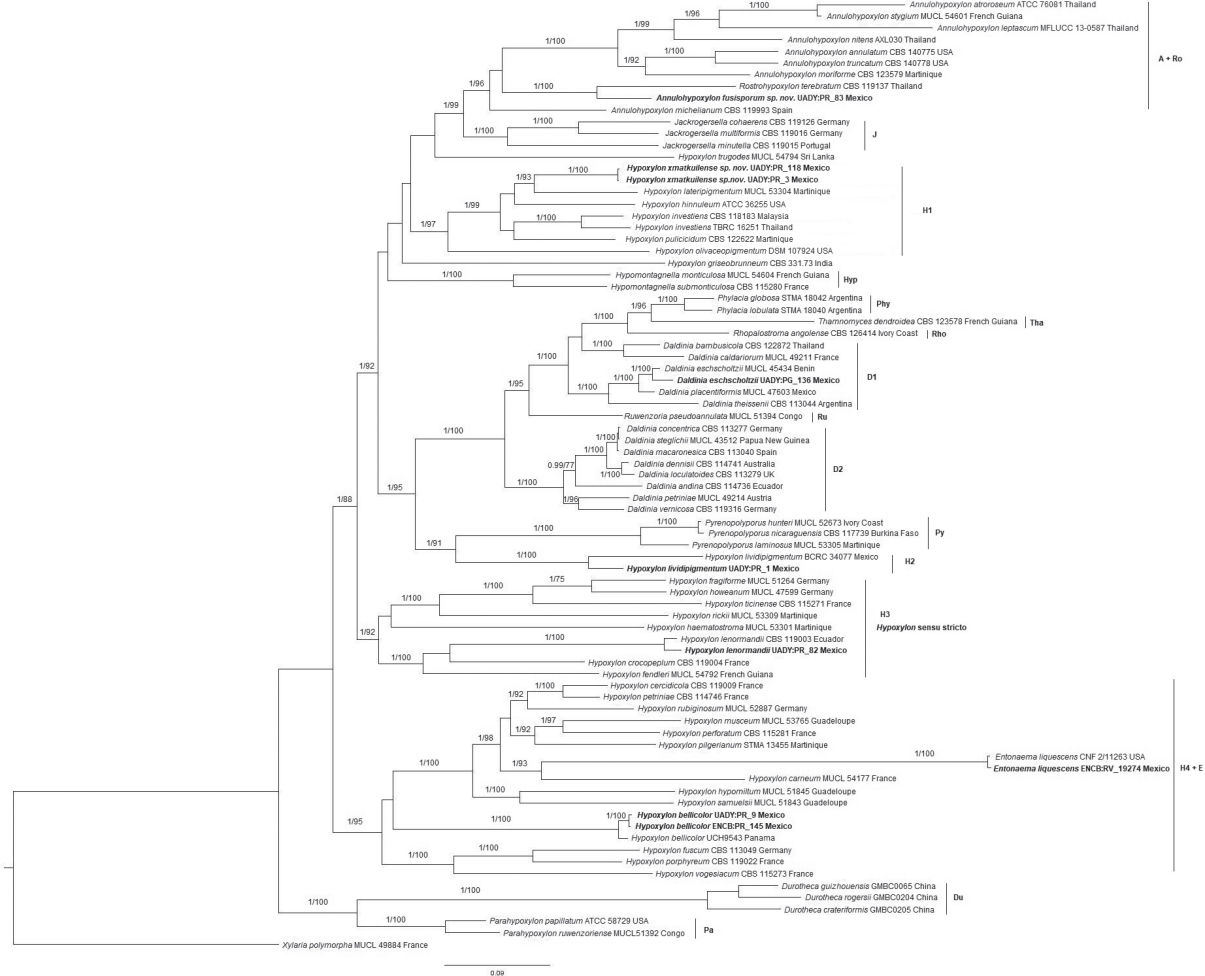
Inferred molecular phylogenetic tree obtained by Bayesian Inference using a multigene alignment (ITS, LSU, RPB2 and TUB2). The tree was rooted with *Xylariapolymorpha*. The sequences generated in the present work and the new combination are highlighted. Bayesian posterior probability values ≥ 0.98 and Bootstrap support values ≥ 70 from the Maximum Likelihood analysis are indicated on the branches.

The family Hypoxylaceae was represented in the phylogenetic tree by nine genera among which we can find: *Phylacia* (Phy) with two specimens, *Thamnomyces* (Tha) one specimen, *Rhopalostroma* (Rho) one specimen, *Daldinia* (D1 and D2) 14 specimens, *Ruwenzoria* (Ru) one specimen, *Pyrenopolyporus* (Py) three specimens, *Annulohypoxylon* (A) nine specimens, *Jackrogersella* three specimens, *Rostrohypoxylon* (Ro) one specimen, *Hypomontagnella* (Hyp) two specimens, *Durotheca* (Du) three specimens, *Parahypoxylon* (Pa) two specimens, *Entonaema* two specimens, *Hypoxylon* (H1, H2, H3, H4) 36 specimens (Fig. 1), which were selected by having molecular information supported by several studies (Wendt et al. 2018; Cedeño-Sanchez et al. 2023; Lambert et al. 2023; Pošta et al. 2023).

In the phylogram resulted from the Bayesian Inference analysis (Fig. 1), *Hypomontagnella*, *Jackrogersella*, *Parahypoxylon*, *Phylacia*, *Pyrenopolyporus*, *Rhopalostroma and Thamnomyces* appear as monophyletic; *Daldinia* as paraphyletic with supports in each group, D1 with 1/95 and D2 with 1/100; *Annulohypoxylon* also paraphyletic with values of 1/96; *Hypoxylon* as polyphyletic in four groups, H1 with values of 1/97, H2 with support of 1/100, H3 with 1/92, H4 + E with 1/95.

The identity of taxa previously reported for the country such as *Daldiniaeschscholtzii*, *Hypoxylonlenormandii*, *H.lividipigmentum* and *Entonaemaliquescens* is confirmed, of which there was limited molecular information for Mexican specimens.

According to the morphological revision of the specimens and the topology of the trees, two new species arise: *Annulohypoxylonfusisporum* and *Hypoxylonxmatkuilense*; the presence of *H.bellicolor* is confirmed in the country.

### ﻿Taxonomy

#### ﻿New species from Mexico

##### ﻿ Annulohypoxylon fusisporum

Taxon classificationFungiXylarialesHypoxylaceae

P. Reyes, Pinzón, R. Valenz. & Raymundo sp. nov.

03BC6F85-A0BB-541E-82D8-B99DB2E67CCC

851003

Fig. 2

###### Gen Bank.

ITS (OR807998), LSU (OR807987), RPB2 (OR825472), TUB2 (OR825468).

###### Diagnosis.

It is characterized by having fusiform spores 10–12 × 4–5 µm, grayish green to dull green KOH-extractable pigments, ¼ perithecial mounds exposed, with straight germ slit in spore-length on the convex side.

###### Etymology.

in reference to the fusiform spores.

###### Holotype.

Mexico • Quintana Roo, Bacalar Experimental Station, Instituto Nacional de Investigaciones Forestales, Agrícolas y Pecuarias; 10 March 2023; P. Reyes leg.; UADY 83.

###### Description.

**Figure 2.**
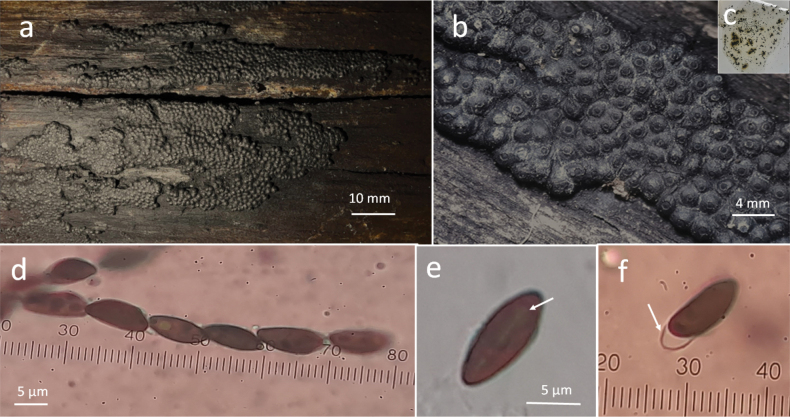
*Annulohypoxylonfusisporum* sp. nov. **a** general view of stromata **b** perithecia spherical with truncatum type ostiolar disc **c** KOH-extractable pigments **d** asci **e** straight germ slit **f** perispore dehiscent 10% KOH.

Stromata effused-pulvinate 20–70 mm long × 20–30 mm wide × 1 mm thick, surface dark brown to black (Fig. 2a) inconspicuous perithecial mounds up to 1/4 exposed (Fig. 2b) dark brown to black granules beneath surface, KOH-extractable pigments Grayish Green (50) changing to Dull Green (70) after 1 minute (Fig. 2c) perithecia 0.7–0.8 mm diam, spherical, conical- papillate ostioles surrounded with a truncatum type disc 0.2–0.25 mm diam; asci 8-spored cylindrical, the spore bearing parts 60–75 µm long × 5–7 µm wide, stipes 20–45 µm long, with amyloid, discoid apical apparatus, 1–2 µm high × 2 µm wide (Fig. 2d); ascospores 10–12 × 4–5 µm, unicellular, fusiform with narrowly rounded ends, with faint, straight germ slit along the spore on the convex side (Fig. 2e), light brown, perispore dehiscent 10% KOH (Fig. 2f).

###### Host.

Growing on dead wood of *Brosimumalicastrum* (Moraceae) in subevergreen forest.

###### Notes.

It is similar to *A.subeffusum* (Hladki and Romero 2009) due to the color, shape of the stromata and the grayish green pigments, however the ascospores of this one are smaller 7–10 × 3–3.8 µm and have inconspicuous perithecial mounds. With *A.leptascum* it shares fusiform shape spores, unlike the length of the germ slit which is short, and the pigments are olive-colored, not changing after 1 minute in 10% KOH; Kuhnert et al. (2016) found BNT, truncatone A and truncatone C in aforementioned taxa.

##### ﻿ Hypoxylon xmatkuilense

Taxon classificationFungiXylarialesHypoxylaceae

P. Reyes, Pinzón, R. Valenz. & Raymundo sp. nov.

57787968-A876-5902-B823-7B91CE54DE36

855643

Fig. 3

###### Gen Bank.

ITS (OR807999), LSU (OR807990), RPB2 (OR825476), TUB2 (OR825467).

###### Diagnosis.

It characterized by having stromata effused-flattened, surface vinaceous, perithecia obovoid and dark brown KOH-extractable pigments.

###### Etymology.

from the Mayan Xmatkuil “place where God is asked” in reference to the place where it was collected for the first time.

###### Holotype.

Mexico • Yucatán, east from cemetery, comisaría Xmatkuil; 14 Oct 2021; P. Reyes leg.; UADY 3.

###### Paratypes.

Mexico • ibid.; 11 July 2023; P. Reyes leg.; UADY 118 • ibid; P. Reyes leg.; UADY 119.

###### Description.

**Figure 3.**
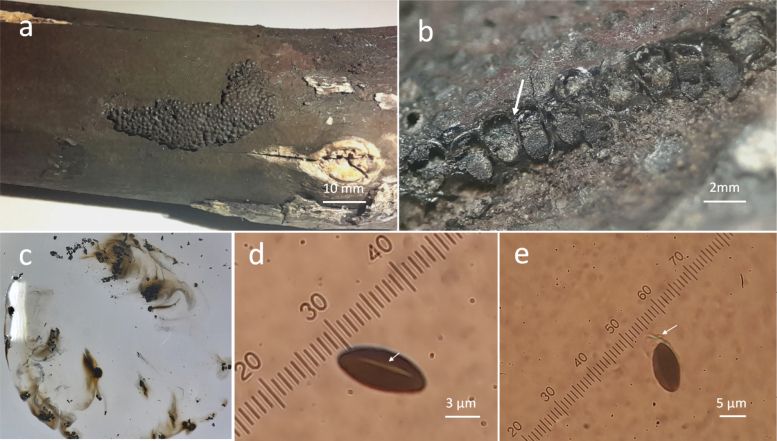
*Hypoxylonxmatkuilense* sp. nov **a** general view of stromata **b** perithecia obovoid **c** KOH-extractable pigments **d** straight germ slit on the convex side **e** perispore dehiscent 10% KOH.

Stromata effused-flattened, 25–100 mm long × 5–20 mm wide × 1 mm thick, surface pruinose, young stromata have a thin layer Dark Vinaceous (82), when mature this layer is lost, leaving remains that appear Brown Vinaceous (84) (Fig. 3a), subsurface blackish, composed of weakly carbonaceous tissue and inconspicuous brownish black granules; perithecia 1.2–1.5 mm diam, obovoid inconspicuous with umbilicate ostioles (Fig. 3b); KOH-extractable pigments dark brown (Fig. 3c); asci not seen; ascospores 10–12 × 4–5 µm, unicellular, ellipsoid, inequilateral, narrowly rounded ends, with straight 2/3 spore-length germ slit on the convex side (Fig. 3d), dark brown, perispore dehiscent in 10% KOH (Fig. 3e).

###### Host.

Growing on dead wood of *Gymnopodiumfloribundum* Rolfe (Polygonaceae) in deciduous forest

###### Notes.

It shares the effused-pulvinate to flattened stromata and vinaceous surface with *H.lateripigmentum*, *H.pulicicidum*, *H.investiens*, *H.hinnuleum*, *H.olivaceopigmentum*, it differs by having perithecia obovoid, dark brown KOH-extractable pigments and perispore dehiscent (Ju and Rogers 1996; Bills et al. 2012; Kuhnert et al. 2014a; Sir et al. 2019); Fournier et al. (2024) described *H.aeneipigmentatum* as a new taxon from Saül, French Guiana, based on ITS sequences, and noted that it belongs *H.investiens* complex; moreover reported about a basal perithecial nucleus as a new differential character in this group.

#### ﻿New record from Mexico

##### ﻿ Hypoxylon bellicolor

Taxon classificationFungiXylarialesHypoxylaceae

Cedeño-Sanchez, L. Wendt & L.C. Mejía 2020 Mycosphere 11(1): 1464 (2020).

9DFD5BB1-BA55-5F5B-B6AC-6E0687AA2775

Fig. 4

###### Description.

**Figure 4.**
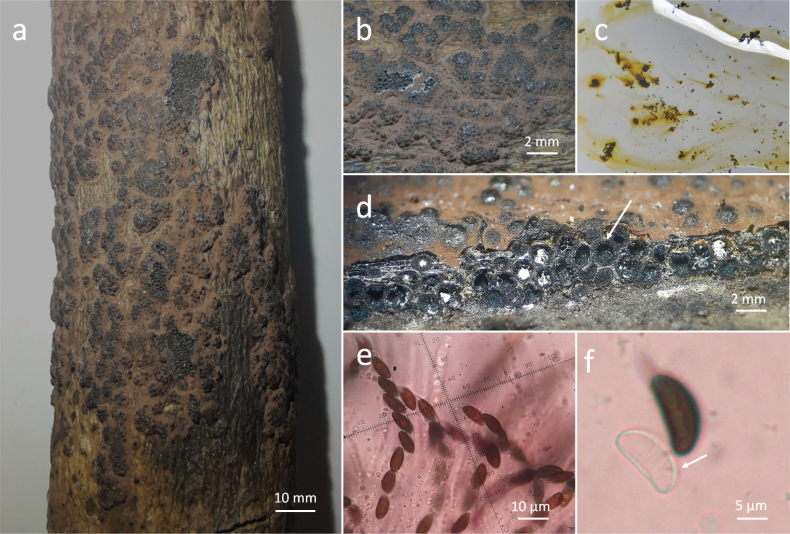
*Hypoxylonbellicolor***a** general view of stromata **b** pruinose surface **c** KOH-extractable pigments **d** perithecia ovoid **e** asci **f** perispore dehiscent 10% KOH with coil-like ornamentation.

Stromata effused-pulvinate 20–90 mm long × 10–30 mm wide, Rust (39) (Fig. 4a) pruinose surface with rust granules beneath the surface (Fig. 4b), KOH-extractable pigments Luteous (12) (Fig. 4c); perithecia ovoid, umbilicate ostioles (Fig. 4d); asci 8-spored cylindrical, the spore bearing part 40–80 µm long × 5–6 µm wide, stipes 30–35 µm long, with amyloid, discoid apical apparatus 2–3 µm high × 2 µm wide (Fig. 4e); ascospores 10–12 µm long × 4–6 µm wide, with germ slit straight less than spore-length on the convex side, perispore dehiscent in 10% KOH with coil-like ornamentation (Fig. 4f).

###### Specimens examined.

Mexico • Campeche, Champotón-Campeche highway; 19 Jan 2018; P. Reyes leg.; UADY 145 • Quintana Roo, Centro de Conservación y Educación Ambiental, Cozumel; 20 Jan 2018; R. Valenzuela leg.; ENCB 17942 •Yucatán, east from the cemetery, comisaría Xmatkuil; 15 Oct 2021; P. Reyes leg.; UADY 7 • ibid.; P. Reyes leg.; UADY 9 •Southern Ecological Park, Mérida; 21 Sept 2022; P. Reyes leg.; UADY 45 • Ría Celestún Biosphere Reserve; 29 Oct 2022; P. Reyes leg.; UADY 55.

###### Host.

Growing on dead wood of *Lysilomalatisiliquum* (L.) Benth. (Fabaceae), *Lonchocarpus* sp. (Fabaceae) in deciduous forest; on dead wood of *Conocarpuserectus* L. (Combretaceae) in coastal dune vegetation.

###### Known distribution.

Panama (Cedeño-Sanchez et al. 2020).

###### Notes.

It shares a stromata color and yellow pigments with *H.perforatum*, however this one is characterized by having a white disc surrounding the ostioles; in addition it has a cosmopolitan distribution (Ju and Rogers 1996).

## ﻿Discussion

Since the segregation of Hypoxylaceae from Xylariaceae (Wendt et al. 2018), many questions have arisen, which have been answered over the years based on the review of groups as *Hypoxylon* that appears as polyphyletic, and from which the genera *Hypomontagnella* (Lambert et al. 2019) and *Parahypoxylon* (Cedeño-Sanchez et al. 2023) have emerged; likewise, *Daldinia* appears grouped in two different clades that are supported in the present study, so it requires a review from integral taxonomy approach to elucidate several questions that will continue to arise within the family.

In the phylogenetic tree of the present study it can be seen that most of the clades of the known genera have good statistical support, *Parahypoxylon*, *Durotheca*, *Jackrogersella*, *Pyrenopolyporus*, *Hypomontagnella*, *Rhopalostroma*, *Thamnomyces*, *Phylacia* and *Ruwenzoria* noted as monophyletic.

As for *Hypoxylon*, it appears as polyphyletic forming four groups (labeled here as H1, H2, H3, and H4, Fig. 1). Group H1 is formed by the following species: 1) *H.investiens* which has a wide distribution, mainly in a tropical climate; it is distinguished by having tubular perithecia. 2) *H.pulicicidum* segregated from the previous one by Fournier et al. (2015b) and Bills et al. (2012) who consider it a rare taxon; it is distinguished by having slightly papillated ostioles and lanceolate perithecia. 3) *H.lateripigmentum* has olive-yellow granules beneath the surface, yellowish brown pigments in 10% KOH and perispore dehiscent. 4) *H.olivaceopigmentum* is distinguished by having the largest spores in the complex 9–15.7 × 4.5–7.2 µm and has been recorded from monocotyledonous hosts. 5) *H.hinnuleum*, proposed by Sir et al. (2019) stands out by having ostioles conical black papillate. Finally, the new species *H.xmatkuilense* is characterized by having perithecia obovoid, dark brown KOH-extractable pigments and perispore dehiscent. This species is sister to *H.lateripigmentum*, which is only known from Martinique.

The members of this group have tropical distribution, *H.xmatkuilense* and *H.lateripigmentum* are Neotropical, so far reported from Caribbean area; *H.olivaceopigmentum* and *H.hinnuleum* from subtropical area, while *H.investiens* and *H.pulicicidum* are Pantropical.

H2 is made up only by *H.lividipigmentum* with two Mexican specimens; this one is characterized by having a sigmoid germ slit in the ascospores, a taxonomic character that is less frequent in the genus.

H3 where the type *H.fragiforme* stands out, so this clade stands as *Hypoxylon* sensu stricto, share distribution with *H.howeanum*, *H.ticinense* of temperate climate, meanwhile *H.crocopeplum*, *H.fendleri*, *H.lenormandii*, *H.haematostroma* and *H.rickii* have been recorded from tropical areas (Wendt et al. 2018; Cedeño-Sanchez et al. 2023).

H4 consists of species of varied distribution and specific plant associations as *H.fuscum* recorded on hosts of the family Betulaceae, in addition to *Acer* and *Salix*; *H.vogesiacum* associated with *Acer*, *H.porphyreum* with *Quercus*; regarding *H.bellicolor*, a new record from Mexico, we registered it growing on Fabaceae and Combretaceae hosts; furthermore, the sequences of the four markers were obtained from two Mexican specimens enriching the knowledge of this taxon, since they were only available ITS and TUB2 sequences (Ju and Rogers 1996; Fournier et al. 2015b; Cedeño-Sanchez et al. 2020).

The polyphyletic origin of *Hypoxylon* encourages more morphological, chemical and phylogenetic studies, including a better sampling of the species to resolve *Hypoxylon* evolutionary history and probably segregate new genera.

On the other hand, our sequences of *Entonaemaliquescens* (ENCB:RV_19274) matched with those of the specimen CNF 2/11263 (Pošta et al. 2023). However, they had no similarity with those from the strain ATCC 46302 (Wendt et al. 2018). This pattern is explained by Stadler et al. (2020) and Cedeño-Sanchez et al. (2024) who point out heterogeneity and polymorphisms among multiple copies of different ITS and LSU loci.

The genus *Daldinia* resulted to be paraphyletic, in agreement with Stadler et al. (2014a), forming two groups; in D1 group, most of the species are exclusively tropical such as *D.bambusicola*, *D.eschscholtzii*, *D.placentiformis* and *D.theissenii*; the D2 group it would be the genus *sensu stricto*, this clade has a widely distribution and there is a recurrence in the hosts of Betulaceae and Lauraceae (Stadler et al. 2014a).

*Annulohypoxylon* also appears as paraphyletic, having *Rostrohypoxylonterebratum* nested inside the clade, which agrees with Tang et al. (2009) and Wendt et al. (2018); it is closely related to the new species *A.fusisporum*, proposed in the current study; Stadler (2011) mentioned that *R.terebratum* represents a lineage that has evolved from *Annulohypoxylon*, while differing in some morphological features and the production of unique secondary metabolites. It is worth mentioning that this taxon could be a turning point for segregating the genus, but more taxonomic and genomic samples are needed.

## ﻿Conclusion

The present study is important because it provides information about the species from a tropical area, particularly from the Yucatan Peninsula, which has a valuable biogeographic history that makes the family have a great diversity, and therefore two new species are described and reports a new record from this region in Mexico; the confirmation of the identity of four species through molecular phylogenetic data is also relevant, since cryptic or semicryptic species may appear, especially in tropical areas.

The proposal of new species is fundamental for the understanding of a recently established family, in which there are still questions to be clarified. These questions will have to be supported by a polyphasic approach that provides comprehensive tools for the interpretation of the phylogenetic relationships in the group.

## Supplementary Material

XML Treatment for Annulohypoxylon fusisporum

XML Treatment for Hypoxylon xmatkuilense

XML Treatment for Hypoxylon bellicolor

## ﻿Additional information

### Author ORCIDs

Pamela E. Reyes https://orcid.org/0000-0003-0882-5245

Juan Pablo Pinzón https://orcid.org/0000-0002-0964-2996

Ricardo Valenzuela https://orcid.org/0000-0001-6596-522

Tania Raymundo https://orcid.org/0000-0002-7525-0034

Juan Tun-Garrido https://orcid.org/0000-0001-8796-3405

Ricardo García-Sandoval https://orcid.org/0000-0001-8641-3305

### Data availability

All of the data that support the findings of this study are available in the main text.

## ﻿Appendix 1

**Table A1.** List of sequences used in phylogenetic analyses obtaneid from Gen Bank. Sequences derived from type material are marked as type (T), holotype (HT), epitype (ET) isotype (IT) and paratype (PT), the sequences obtained in this study are highlighted.

Species	Strain/voucher number	Origin and status	ITS	LSU	RPB2	TUB2	Reference
***Annulohypoxylonannulatum***	CBS 140775	USA (ET)	KY610418	MK287546	KY624263	KX376353	(Kuhnert et al. 2016; Wendt et al. 2018)
***Annulohypoxylonatroroseum***	ATCC 76081	Thailand	AJ390397	KY610422	KY624233	DQ840083	(Kuhnert et al. 2014a; Wendt et al. 2018)
***Annulohypoxylonfusisporum* sp. nov.**	**UADY:PR_83**	**Mexico (HT)**	**OR807998**	**OR807987**	**OR825472**	**OR825468**	**This study**
***Annulohypoxylonleptascum***	MFLUCC 13-0587	Thailand	KU604576			KU604580	Sir et al. (2016a)
***Annulohypoxylonmichelianum***	CBS 119993	Spain	KX376320	KY610423	KY624234	KX271239	Wendt et al. (2018); Kuhnert et al. (2014a)
***Annulohypoxylonmoriforme***	CBS 123579	Martinique	KX376321	KY610425	KY624289	KX271261	Wendt et al. (2018); Kuhnert et al. (2016)
***Annulohypoxylonnitens***	AXL030	Thailand	KJ934991	KJ934992	KJ934994	KJ934993	Daranagama et al. (2015)
***Annulohypoxylonstygium***	MUCL 54601	French Guiana	KY610409	KY610475	KY624292	KX271263	Wendt et al. (2018)
***Annulohypoxylontruncatum***	CBS 140778	USA (ET)	KY610419	KY610419	KY624277	KY624277	Wendt et al. (2018); Kuhnert et al. (2016)
***Daldiniaandina***	CBS 114736	Ecuador (HT)	AM749918	KY610430	KY624239	KC977259	Bitzer et al. (2008)
***Daldiniabambusicola***	CBS 122872	Thailand (HT)	KY610385	KY610431	KY624241	KY624241	Wendt et al. (2018); Hsieh et al. (2005)
***Daldiniacaldariorum***	MUCL 49211	France	AM749934	KY610433	KY624242	KC977282	Wendt et al. (2018); Kuhnert et al. (2014a); Bitzer et al. (2008)
***Daldiniaconcentrica***	CBS 113277	Germany	AY616683	KY610434	KY624243	KC977274	Wendt et al. (2018); Kuhnert et al. (2014a); Triebel et al. (2005)
***Daldiniadennisii***	CBS 114741	Australia (HT)	JX658477	KY610435	KY624244	KC977262	Stadler et al. (2014a); Kuhnert et al. (2014a); Wendt et al. (2018)
***Daldiniaeschscholtzii***	MUCL 45434	Benin	JX658484	KY610437	KY624246	KC977266	Stadler et al. (2014a); Kuhnert et al. (2014a); Wendt et al. (2018)
***Daldiniaeschscholtzii***	**UADY:PG_136**	**Mexico**	**OR808001**	**OR807988**	**OR825473**	**OR825469**	**This study**
***Daldinialoculatoides***	CBS 113279	UK (ET)	AF176982	KY610438	KY610438	KX271246	Johannesson et al. (2000); Wendt et al. (2018)
***Daldiniamacaronesica***	CBS 113040	Spain (PT)	KY610398	KY610477	KY624294	KX271266	Wendt et al. (2018)
***Daldiniapetriniae***	MUCL 49214	Austria (ET)	AM749937	KY610439	KY624248	KC977261	Bitzer et al. (2008); Kuhnert et al. (2014a); Wendt et al. (2018)
***Daldiniaplacentiformis***	MUCL 47603	Mexico	AM749921	KY610440	KY624249	KC977278	Bitzer et al. (2008); Kuhnert et al. (2014a); Wendt et al. (2018)
***Daldiniasteglichii***	MUCL 43512	Papua New Guinea (PT)	KY610399	KY610479	KY624250	KX271269	Wendt et al. (2018)
***Daldiniatheissenii***	CBS 113044	Argentina (PT)	KY610388	KY610441	KY624251	KX271247	Wendt et al. (2018)
***Daldiniavernicosa***	CBS 119316	Germany (PT)	KY610395	KY610442	KY624252	KC977260	Kuhnert et al. (2014a); Wendt et al. (2018)
***Durothecacrateriformis***	GMBC0205	China (T)	MH645426	MH645425	MH645427	MH049441	De Long et al. (2019)
***Durothecaguizhouensis***	GMBC0065	China (T)	MH645423	MH645421	MH645422	MH049439	De Long et al. (2019)
***Durothecarogersii***	GMBC0204	China	MH645433	MH645434	MH645435	MH049449	De Long et al. (2019)
***Entonaemaliquescens***	CNF 2/11263	USA	OQ869784.1	OQ865124.1	OQ877106.1	OQ877117.1	Pošta et al. (2023)
***Entonaemaliquescens***	**ENCB:RV_19274**	**Mexico**	**OR807997**	**OR807993**	**OR825474**	**OR825466**	**This study**
***Hypoxylonbellicolor***	UCH9543	Panama	MN056425.1			MK908139	Cedeño-Sanchez et al. (2020)
***Hypoxylonbellicolor***	**UADY:PR_9**	**Mexico**	**OR808002**	**OR807989**		**OR825470**	**This study**
***Hypoxylonbellicolor***	**ENCB:PR_145**	**Mexico**	**OR808004**	**OR807994**	**OR825475**	**OR825471**	**This study**
***Hypoxyloncarneum***	MUCL 54177	France	KY610400	KY610480	KY624297	KX271270	Wendt et al. (2018)
***Hypoxyloncercidicola***	CBS 119009	France	KC968908	KY610444	KY610444	KC977263	Kuhnert et al. (2014a); Wendt et al. (2018)
***Hypoxyloncrocopeplum***	CBS 119004	France	KC968907	KY610445	KY624255	KC977268	Kuhnert et al. (2014a); Wendt et al. (2018)
***Hypoxylonfendleri***	MUCL 54792	French Guiana	KF234421	KY610481	KY624298	KF300547	Kuhnert et al. (2014a); Wendt et al. (2018)
***Hypoxylonfragiforme***	MUCL 51264	Germany (ET)	KC477229	KM186295	KM186296	KM186296	Stadler et al. (2013); Daranagama et al. (2015); Wendt et al. (2018)
***Hypoxylonfuscum***	CBS 113049	Germany (ET)	KY610401	KY610482	KY624299	KX271271	Wendt et al. (2018)
***Hypoxylongriseobrunneum***	CBS 331.73	India (HT)	KY610402	KY610483	KY624300	KC977303	Kuhnert et al. (2014a); Wendt et al. (2018)
***Hypoxylonhaematostroma***	MUCL 53301	Martinique (ET)	KC968911	KY610484	KY624301	KC977291	Kuhnert et al. (2014a); Wendt et al. (2018)
***Hypoxylonhinnuleum***	ATCC 36255	USA (T)	MK287537	MK287549	MK287562	MK287575	Sir et al. (2019)
***Hypoxylonhoweanum***	MUCL 47599	Germany	AM749928	KY610448	KY624258	KC977277	Bitzer et al. (2008); Kuhnert et al. (2014a); Wendt et al. (2018)
***Hypoxylonhypomiltum***	MUCL 51845	Guadeloupe	KY610403	KY610449	KY624302	KX271249	Wendt et al. (2018)
***Hypoxyloninvestiens***	CBS 118183	Malaysia	KC968925	KY610450	KY624259	KC977270	Kuhnert et al. (2014a); Wendt et al. (2018)
***Hypoxyloninvestiens***	TBRC 16251	Thailand			OQ108848.1	OQ144968.1	Suetrong et al. (2023)
***Hypoxylonlateripigmentum***	MUCL 53304	Martinique (HT)	KC968933	KY610486	KY624304	KC977290	Kuhnert et al. (2014a); Wendt et al. (2018)
***Hypoxylonlenormandii***	CBS 119003	Ecuador	KC968943	KY610452	KY624261	KC977273	Kuhnert et al. (2014a); Wendt et al. (2018)
***Hypoxylonlenormandii***	**UADY:PR_82**	**Mexico**	**OR808003**	**OR807991**	**OR825477**		**This study**
***Hypoxylonlividipigmentum***	BCRC 34077	Mexico (IT)	JN979433			AY951735	Hsieh et al. (2005)
***Hypoxylonlividipigmentum***	**UADY:PR_1**	**Mexico**	**OR808000**	**OR807992**	**OR825478**		**This study**
***Hypomontagnellamonticulosa***	MUCL 54604	French Guiana (ET)	KY610404	KY610487	KY624305	KX271273	Wendt et al. (2018)
***Hypoxylonmusceum***	MUCL 53765	Guadeloupe	KC968926	KY610488	KY624306	KC977280	Kuhnert et al. (2014a); Wendt et al. (2018)
***Hypoxylonolivaceopigmentum***	DSM 107924	USA (T)	MK287530	MK287542	MK287555	MK287568	Sir et al. (2019)
***Hypoxylonperforatum***	CBS 115281	France	KY610391	KY610455	KY624224	KY624224	Wendt et al. (2018)
***Hypoxylonpetriniae***	CBS 114746	France (HT)	KY610405	KY610491	KY624279	KX271274	Kuhnert et al. (2016); Wendt et al. (2018)
***Hypoxylonpilgerianum***	STMA 13455	Martinique	KY610412	KY610412	KY624308	KY624315	Wendt et al. (2018)
***Hypoxylonporphyreum***	CBS 119022	France	KC968921	KY610456	KY624225	KC977264	Kuhnert et al. (2014a); Wendt et al. (2018)
***Hypoxylonpulicicidum***	CBS 122622	Martinique (HT)	JX183075	KY610492	KY624280	JX183072	Bills et al. (2012); Wendt et al. (2018)
***Hypoxylonrickii***	MUCL 53309	Martinique (ET)	KC968932	KY610416	KY624281	KC977288	Kuhnert et al. (2014a); Wendt et al. (2018)
***Hypoxylonrubiginosum***	MUCL 52887	Germany (ET)	KC477232	KY610469	KY624266	KY624311	Stadler et al. (2013); Wendt et al. (2018)
***Hypoxylonsamuelsii***	MUCL 51843	Guadeloupe (ET)	KC968916	KY610466	KY624269	KC977286	Kuhnert et al. (2014a); Wendt et al. (2018)
***Hypomontagnellasubmonticulosa***	CBS 115280	France	KC968923	KY610457	KY624226	KC977267	Kuhnert et al. (2014a); Wendt et al. (2018)
***Hypoxylonticinense***	CBS 115271	France	JQ009317	KY610471	KY624272	AY951757	Hsieh et al. (2005); Wendt et al. (2018)
***Hypoxylontrugodes***	MUCL 54794	Sri Lanka (ET)	KF234422	KY610493	KY624282	KF300548	Kuhnert et al. (2014a); Wendt et al. (2018)
***Hypoxylonvogesiacum***	CBS 115273	France	KC968920	KY610417	KY624283	KX271275	Kuhnert et al. (2014a); Wendt et al. (2018)
***Hypoxylonxmatkuilense* sp.nov.**	**UADY:PR_3**	**Mexico (HT)**	**OR807999**	**OR807990**	**OR825476**	**OR825467**	**This study**
***Hypoxylonxmatkuilense* sp. nov.**	**UADY:PR_118**	**Mexico (PT)**	**OR807996**	**OR807995**	**PP239283**	**PP239284**	**This study**
***Jackrogersellacohaerens***	CBS 119126	Germany	KY610396	KY610497	KY624270	KY624314	Wendt et al. (2018)
***Jackrogersellaminutella***	CBS 119015	Portugal	KY610381	KY610424	KY610424	KX271240	Kuhnert et al. (2016); Wendt et al. (2018)
***Jackrogersellamultiformis***	CBS 119016	Germany (ET)	KC477234	KY610473	KY624290	KX271262	Kuhnert et al. (2014a); Wendt et al. (2018)
***Parahypoxylonpapillatum***	ATCC 58729	USA (T)	KC968919	KY610454	KY624223	KC977258	Kuhnert et al. (2014a), Wendt et al. (2018)
***Parahypoxylonruwenzoriense***	MUCL51392	Congo (T)	ON792786	ON954156	OP251039	ON813078	Cedeño-Sanchez et al. (2023)
***Phylaciaglobosa***	STMA 18042	Argentina	OQ437889.1	OQ437885	OQ453168	OQ453172	Lambert et al. (2023)
***Phylacialobulata***	STMA 18040	Argentina	OQ437893.1	OQ437883	OQ453164	OQ453170	Lambert et al. (2023)
***Pyrenopolyporushunteri***	MUCL 52673	Ivory Coast (ET)	KY610421	KY610472	KY624309	KU159530	Kuhnert et al. (2016); Wendt et al. (2018)
***Pyrenopolyporuslaminosus***	MUCL 53305	Martinique (HT)	KC968934	KY610485	KY624303	KC977292	Kuhnert et al. (2014a); Wendt et al. (2018)
***Pyrenopolyporusnicaraguensis***	CBS 117739	Burkina Faso	AM749922	KY610489	KY624307	KC977272	Bitzer et al. (2008); Wendt et al. (2018)
***Rhopalostromaangolense***	CBS 126414	Ivory Coast	KY610420	KY610459	KY624228	KX271277	Wendt et al. (2018)
***Rostrohypoxylonterebratum***	CBS 119137	Thailand (HT)	DQ631943	DQ840069	DQ631954	DQ840097	Tang et al. (2007); Fournier et al. (2010)
***Ruwenzoriapseudoannulata***	MUCL 51394	D.R. Congo (HT)	KY610406	KY610494	KY624286	KX271278	Wendt et al. (2018)
***Thamnomycesdendroidea***	CBS 123578	French Guiana (HT)	FN428831	KY610467	KY624232	KY624313	Stadler et al. (2010b); Wendt et al. (2018)
***Xylariapolymorpha***	MUCL 49884	France	KY610408	KY610464	KY624288	KX271280	Wendt et al. (2018)
